# Evaluation of Epidemiological Cut-Off Values Indicates that Biocide Resistant Subpopulations Are Uncommon in Natural Isolates of Clinically-Relevant Microorganisms

**DOI:** 10.1371/journal.pone.0086669

**Published:** 2014-01-23

**Authors:** Ian Morrissey, Marco Rinaldo Oggioni, Daniel Knight, Tania Curiao, Teresa Coque, Ayse Kalkanci, Jose Luis Martinez, the BIOHYPO Consortium

**Affiliations:** 1 Quotient Bioresearch, Fordham, United Kingdom; 2 Dipartimento de Biotecnologie, Università di Siena, Siena, Italy; 3 Servicio de Microbiología and CIBER en Epidemiología y Salud Pública and Instituto Ramón y Cajal de Investigación Sanitaria, Madrid, Spain; 4 Gazi University School of Medicine, Department of Medical Microbiology, Ankara, Turkey; 5 Centro Nacional de Biotecnologia–CSIC, Madrid, Spain; University of Padova, Medical School, Italy

## Abstract

To date there are no clear criteria to determine whether a microbe is susceptible to biocides or not. As a starting point for distinguishing between wild-type and resistant organisms, we set out to determine the minimal inhibitory concentration (MIC) and minimal bactericidal concentration (MBC) distributions for four common biocides; triclosan, benzalkonium chloride, chlorhexidine and sodium hypochlorite for 3319 clinical isolates, with a particular focus on *Staphylococcus aureus* (N = 1635) and *Salmonella* spp. (N = 901) but also including *Escherichia coli* (N = 368), *Candida albicans* (N = 200), *Klebsiella pneumoniae* (N = 60), *Enterobacter* spp. (N = 54), *Enterococcus faecium* (N = 53), and *Enterococcus faecalis* (N = 56). From these data epidemiological cut-off values (ECOFFs) are proposed. As would be expected, MBCs were higher than MICs for all biocides. In most cases both values followed a normal distribution. Bimodal distributions, indicating the existence of biocide resistant subpopulations were observed for *Enterobacter* chlorhexidine susceptibility (both MICs and MBCs) and the susceptibility to triclosan of *Enterobacter* (MBC), *E. coli* (MBC and MIC) and *S. aureus* (MBC and MIC). There is a concern on the potential selection of antibiotic resistance by biocides. Our results indicate however that resistance to biocides and, hence any potential association with antibiotic resistance, is uncommon in natural populations of clinically relevant microorganisms.

## Introduction

Biocides have been used extensively for decades and are present in a wide range of commonly used compounds, including pesticides, disinfectants, antiseptics, preservatives for food, antifouling products, toothpastes, home-used detergents and even at some formulations of concrete or textiles among others [1]–[3]. The increased use of biocides for an expanding range of applications have raised concerns on the potential effect their use may have for human health as well as on the impact on the natural populations of microorganisms [1], [4]–[6]. In particular, there is growing concern regarding the possible effect the widespread use of biocides may have on selection for antibiotic resistance in clinically relevant microorganisms [7]–[12]. The fact that many formulations/products contain biocides at low concentrations and that the discharge of biocides in natural ecosystems produce a full landscape of biocide selective concentrations might enhance the risk of selection of resistant microbes. In 2009, the Scientific Committee on Emerging and Newly Identified Health Risks (SCENHIR) produced a report for the European Commission entitled Assessment of the Antibiotic Resistance Effects of Biocides (http://ec.europa.eu/health/ph_risk/committees/04_scenihr/docs/scenihr_o_021.pdf). In this report, it was stated that ‘biocides are likely to contribute to maintaining selective pressure allowing the presence of mobile genetic elements harboring specific genes involved in the resistance to biocides and antibiotics’. One recommendation of the SCENHIR report was to have standardized methodologies and surveillance programs to monitor levels of biocide resistance.

Current methodologies for studying the effect of biocides are based on the evaluation of the potency of the compound itself for killing an organism in a short time lapse. Since the methods are based on the analysis of the compound and not on the study of the microorganisms, there is not a clear definition of biocide resistance. Differing to the situation with most antibiotics where clinical outcome data, PK/PD models and MIC distributions are used to determine clinical breakpoints (susceptible, intermediate or resistant) to guide therapy [13], a similar definition of biocide resistance, based on breakpoints, is absent. All previous studies dealing with this topic, compare the MIC (measured using same methods as those used for antibiotics) of a wild-type strain with another isolate containing a mutation or a gene presumptively encoding biocide resistance. If the second is less susceptible than wild-type, it is considered as resistant. This method can be of value when the potentially resistant strain derives from the wild-type one, which is considered as susceptible by definition. However, it is of no use when natural isolates are studied, because as is stated above there are not breakpoints to define resistance to biocides and consequently such isolates cannot be classified as susceptible or resistant. Consequently, whereas *in vitro* work on the role of biocide resistance on the selection of antibiotic resistance has produced solid results [7], [8], [14]–[16], the absence of a definition of biocide resistance has meant that available epidemiological data in this respect is extremely limited [4], [16].

The aim of the present work is to establish appropriate breakpoints for defining biocide resistance for those biocides as triclosan (TRI), benzalkonium chloride (BZC), chlorhexidine (CHX) and hypochloride for which more concerns on the potential coselection of antibiotic resistance have been raised. These breakpoints will be the hallmarks for future studies to define mechanisms of biocide resistance as well as for analyzing the potential selection of antibiotic resistance by biocides in natural isolates. For this purpose, we have made use of the concept of epidemiological cut-off values (ECOFFs, http://www.eucast.org/fileadmin/src/media/PDFs/EUCAST_files/EUCAST_Presentations/2011/EW1_Brown_Definitionsf2.pdf). These breakpoints are not based, as clinical breakpoints are, on the likelihood of treatment failure to define resistance, rather ECOFFs are defined on the basis of the normal distribution of MICs in a given bacterial species. All isolates which have MICs inside this distribution are considered as wild-type, and those presenting MICs above this value are considered as resistant [6]. Reference MIC distributions and ECOFFs for many microorganism-antibiotic combinations are collated by the European Committee on Antimicrobial Susceptibility Testing (EUCAST) to help highlight those organisms that may have acquired resistance mechanisms (http://www.eucast.org/mic_distributions/).

In this study, therefore, we evaluated MIC distributions for TRI, BZC, CHX and NaOCl against 3327 clinical isolates belonging to relevant microbial pathogen species. Minimal bactericidal/fungicidal concentrations (MBC/MFC) distributions were also evaluated to take into account the microbicidal properties of biocides. Based on these distributions, ECOFFs (for MIC and MBC) are proposed for these biocides to assist future surveillance of biocide susceptibility and also help discover potential resistance mechanisms to biocides.

## Materials and Methods

### Bacterial and Fungal Strains

The following strains were evaluated and came from strain collections held at Quotient Bioresearch (Fordham, UK), Hospital Universitario Ramón y Cajal (Madrid, Spain) and Gazi University School of Medicine (Ankara, Turkey). 1635 *Staphylococcus aureus* strains collected between 2002 and 2003, from different geographical origins (world-wide), representing both hospital and community acquired infections; 901 *Salmonella* spp. collected between 1999 and 2003 from European veterinary sources; 368 *Escherichia coli* collected between 1998 and 2011 from Spain; 200 *Candida albicans* collected in 2010 and 2011 from hospital acquired infections and vulvovaginal candidiasis in Turkey; 50 *Klebsiella pneumoniae* collected between 1991 and 2011 from Spain; 53 *Enterococcus faecium* collected between 1986 and 2009 from world-wide locations; 56 *Enterococcus faecalis* collected between 2001 and 2009 from Spain and 54 Enterobacter spp. collected between 1991–2011 from Spain.

### Biocides

Stock solutions of Benzalkonium chloride (BZC; Sigma, B6295) and Chlorhexidine digluconate (CHX; Sigma, C9394) were prepared in sterile distilled water at a concentration of 100 mg/ml prior to further dilution in broth and distribution into 96-well plates. Stock solutions of triclosan (TRI; Irgasan; Sigma 72779) were prepared at 400 mg/L in methanol and a dilution series at 100x final concentration prepared in methanol prior to 1∶100 dilution in broth in 96-well plates. Sodium hypochlorite (Sigma, 425044) was prepared in sterile distilled water at a concentration range between 0.128–65 g/L at serial dilutions in 96-well plates. All solutions were freshly prepared on the day of the experiment and kept protected from light.

### Susceptibility Testing

Minimum inhibitory concentrations (MICs) were determined using the broth microdilution method set by the Clinical Laboratory Standards Institute (CLSI) [13]. Determination of the minimum bactericidal concentrations (MBCs) or minimum fungicidal concentrations (MFCs) were performed by subculturing 10 µl from each well without visible microbial growth. After 48 hours of incubation the biocide dilution yielding three colonies or less was scored as the MBC/MFC as described by the CLSI for starting inocula of 1×10^5^ CFU/ml [14].

### Determination of ECOFFs

Where unimodal MIC or MBC/MFC distributions were shown ECOFFs were determined as concentrations representing ≥99.9% of the bacterial population (MIC_99.9_, MBC_99.9_ or MFC_99.9_), as described previously [15]. If the distribution was bimodal the ECOFF was set between the two populations.

## Results and Discussion

To date there are no clear criteria to determine whether a given microbe non-susceptible to biocides or not. Even in the case of antibiotic resistance, different definitions have been proposed [17]. As stated by EUCAST (http://www.eucast.org/fileadmin/src/media/PDFs/EUCAST_files/EUCAST_Presentations/2011/EW1_Brown_Definitionsf2.pdf), from the clinical point of view “a microorganism is defined as susceptible by a level of antimicrobial activity associated with a high likelihood of therapeutic success” and “a microorganism is defined as resistant by a level of antimicrobial activity associated with a high likelihood of therapeutic failure”. These operational definitions of resistance do not discriminate between wild-type organisms and those that have acquired low-level resistance to antibiotics if the MICs achieved still categorize these bacteria as susceptible. In the case of biocides, these definitions are not useful, because these compounds are frequently used at surfaces, where their concentrations can be much higher than in the case of therapeutic agents, for which toxicity and pharmacodynamics constrain the actual concentrations faced by the microorganisms during treatment. As the consequence of this situation there is not currently any operational definition of resistance in the case of biocides, which makes difficult performing meaningful epidemiological analysis on biocide resistance in natural bacterial isolates. Furthermore, comparing results from different studies is also made very difficult.

In order to discuss biocide resistance, we require a more suited definition, one which is based on the “natural” susceptibility to antimicrobials of a given species and not just on the clinical success of the treatment. This ecological concept of resistance states that “a microorganism is defined as wild type for a species by the absence of acquired and mutational mechanisms of resistance to the agent” (http://www.eucast.org/fileadmin/src/media/PDFs/EUCAST_files/EUCAST_Presentations/2011/EW1_Brown_Definitionsf2.pdf). The definition of the wild-type MIC phenotype is obtained by the study of several unrelated isolates, which allow establishing the epidemiological cut-off value (ECOFF), which is the upper limit of the normal MICs distribution for a given antimicrobial and a given species. Any isolate presenting a MIC above this value is considered as resistant irrespective of whether or not the achieved level of resistance compromises therapy.

**Figure 1:**
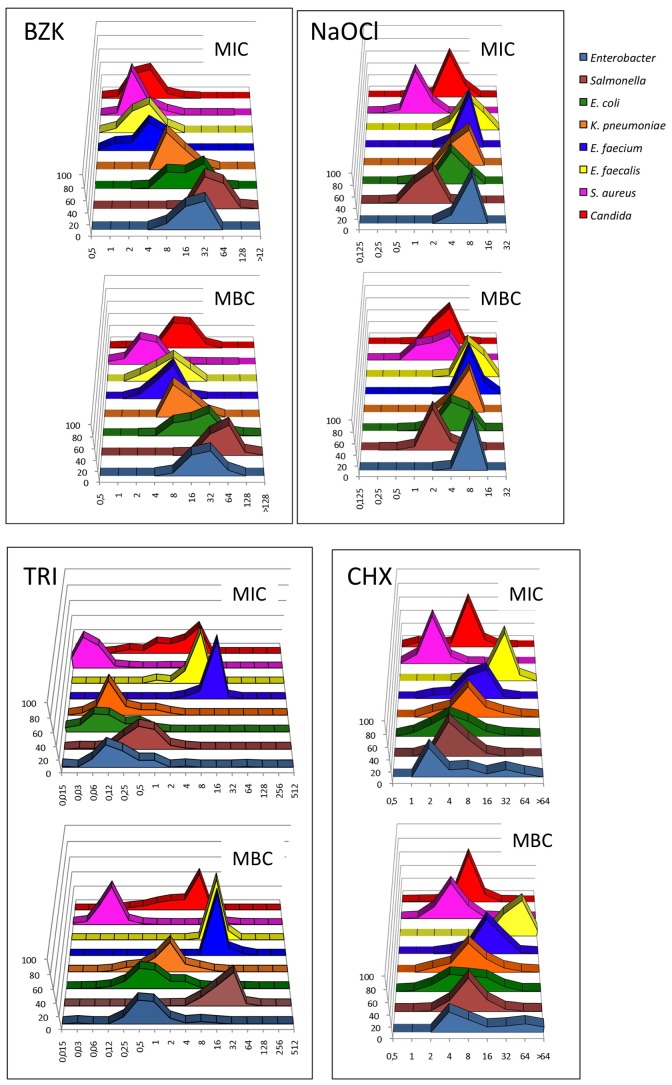
Populational susceptibility to biocides of different pathogens: To analyze the overall susceptibility to the studied biocides, the MICs and MBCs (MFCs in the case of Candida) of several independent isolates were established. The name of the studied biocide is displayed in each panel. In all cases the upper graph shows the MICs distributions and the lower one the MBCs distributions. TRI, CHX and BZC, concentration as expressed in mg/L. For Nalco concentration is expressed as g/L of active chlorine.

As a starting point for distinguishing between wild-type and resistant organisms, we set out to determine the distributions of the MICs and the MBCs of TRI, BZC, CHX and NaOCl for natural isolates of different relevant pathogens. We name “natural isolates” as those that have been isolated from any habitat (including infection), but have not been sub-cultured for several generations under laboratory-growing conditions. The tested organisms included *“bacterial species submitted to selective pressure, involved in the transmission of MGE and directly involved in the biological hazard (final host)”* as recommended by the Scientific Committee on Emerging and Newly Identified Health Risks (SCENIHR; http://ec.europa.eu/health/ph_risk/committees/04_scenihr/docs/scenihr_o_021.pdf Among them, we focused on isolates representing main clonal lineages and mobile genetic elements involved in spread of antibiotic resistance (*Enterobacteriaceae*, *Enterococci*) and isolates of foodborne and zoonotic pathogens (*S. aureus, Salmonella*). *Candida* isolates were also included as representative of fungi of medical and veterinary relevance. To avoid over-representation of epidemic clones that could produce a bias on the studied bacterial populations, isolates had different geographical and/or temporal origins. As would be expected, MBCs were higher than MICs for all biocides and in most cases both values followed a normal distribution (Figure 1), with some few exceptions that will be discussed later on. This type of MIC distribution is the expected one for non-biased bacterial populations, which indicates that our samples are appropriate for defining MIC and MBC ECOFF values for the tested biocides and populations. Using this information, MIC_50_/MBC_50_ and MIC_90_/MBC_90_ values for each of the analyzed species were determined (Table 1) as well as the MIC and MBC ECOFF values (Table 2). As shown in the Table 1 and Figure 1, *S. aureus* was the most susceptible species to all tested biocides and Enterococci the least susceptible organism group for TRI, CHX and NaOCl, whereas *K. pneumoniae*, *E. coli*, *Salmonella* and *Enterobacter* spp., presented the lowest susceptibilities to BZC.

**Table 1:** MIC50 and MIC90 of four common biocides for 3327 microbial isolates.

Species	N^a^	TRI^b^				CHX^b^				BZC^b^				NaOCl^b^			
		MIC (0.015–128)^c^		MBC (0.015–512)^c^		MIC (0.5–64)^c^		MBC (0.5–64)^c^		MIC (0.5–128)^c^		MBC (0.5–128)^c^		MIC (0.125–32)		MBC (0.125–32)	
		50	90	50	90	50	90	50	90	50	90	50	90	50	90	50	90
***Salmonella*** ** spp.**	901	0.06	0.12	1	2	32	64	64	64	16	16	16	32	2	2	2	4.1
***E. coli***	368	0.12	0.5	1	4	4	16	8	16	16	32	16	32	4.1	4.1	8.2	8.2
***K. pneumoniae***	60	0.12	1	1	2	8	32	8	>32	8	16	8	16	8.2	8.2	8.2	8.2
***Enterobacter*** ** spp.**	54	0.12	0.5	1	2	8	64	8	64	16	32	32	64	4.1	4.1	4.1	4.1
***S. aureus***	1635	0.03	0.06	0.12	0.25	2	4	4	8	2	4	2	4	1	2	2	4.1
***E. faecium***	53	8	8	16	16	8	16	16	32	4	8	4	8	8.2	8.2	8.2	16.4
***E. faecalis***	56	8	8	16	16	32	32	32	64	2	4	8	8	4.1	8.2	4.1	8.2
***C. albicans***	200	4	8	8	8	8	8	8	16	4	4	8	16	4.1	4.1	4.1	8.2

a N: number of strains.

b TRI, CHX and BZC, concentration as expressed in mg/L. For NaOCl concentration is expressed as g/L of active chlorine.

c Range of tested concentrations. TRI, CHX and BZC, concentration as expressed in mg/L. For NaOCl concentration is expressed as g/L of active chlorine.

**Table 2:** MIC and MBC ECOFFs of four common biocides for 3327 microbial isolates.

Species	N*	TRI**		CHX**		BZC**		NaOCl**	
		MIC	MBC	MIC	MBC	MIC	MBC	MIC	MBC
***Salmonella*** ** spp.**	901	8	128	32	>64	128	>128	4.1	8.2
***E. coli***	368	2	16	64	>64	64	128	8.2	16.4
***K. pneumoniae***	60	2	8	64	64	32	32	8.2	8.2
***Enterobacter*** ** spp.**	54	1	4	16	16	32	64	4.1	8.2
***S. aureus***	1635	0.5	2	8	>64	16	32	4.1	8.2
***E. faecium***	53	32	64	32	64	8	16	8.2	16.4
***E. faecalis***	56	16	32	64	>64	8	16	8.2	8.2
***C. albicans***	200	16	16	16	32	16	32	8.2	16.4

* N: number of strains.

** TRI, CHX and BZC, concentration as expressed in mg/L. For NaOCl concentration is expressed as g/L of active chlorine.

In the cases in which the ECOFFs for antibiotics have been studied, the MICs of the populations frequently follow bimodal of even multimodal distributions. These distributions indicate the existence of different subpopulations, each one with a different level of susceptibility due to the presence of specific mechanisms of resistance. In this current study, bimodal distributions were uncommon and observed for just *Enterobacter* CHX susceptibility (both MICs and MBCs), TRI susceptibility to of *Enterobacter* (MBC), *E. coli* (MBC and MIC) and *S. aureus* (MBC and MIC). For *S. aureus* those with high TRI MBC were found all to harbor either a mutated *fabI* gene, a mutation in the *fabI* promoter or an added *fabI* gene [18]. None of the screened phenotypically susceptible strains harbored any of these markers [18]. This indicates that TRI resistance is due to either changes in the in-host FabI, either to the acquisition of a second *fabI* copy in the case of *S. aureus*. None of these TRI resistance mechanisms correlates with an increase in antibiotic resistance. *Enterobacteriaceae* were tested for genes previously associated with biocide resistance (*qac*, *acrAB*, *fabI*). Surprisingly, reduced susceptibility to BZC was not linked to the presence of *qac* genes *qacI*, *qacE* and *qacK* despite these genes appear in 5–20% of the *E. coli* isolates analyzed. Similarly to the situation observed for *S. aureus*, changes in the *fabI* sequences were noted for *E. coli* isolates with reduced susceptibility to TRI (TC *et al.* to be published).

**Figure 2:**
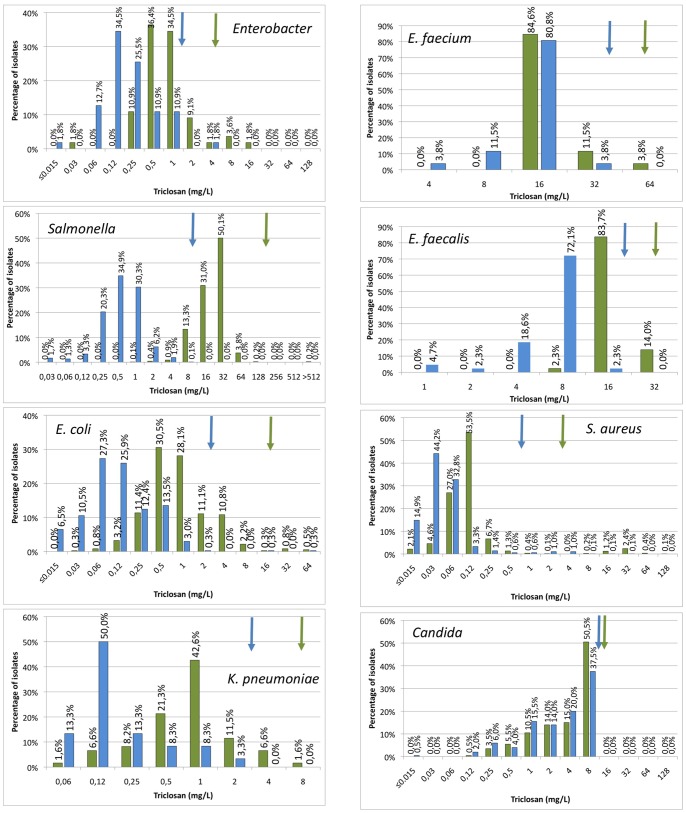
Populational susceptibility to triclosan of different pathogens: To analyze the overall susceptibility to triclosan, the MICs and MBCs (MFCs in the case of Candida) of several independent isolates were established. Blue bars MICs, green bars MBCs/MFCs. ECOFFs are shown with arrows of the corresponding colour.

**Figure 3:**
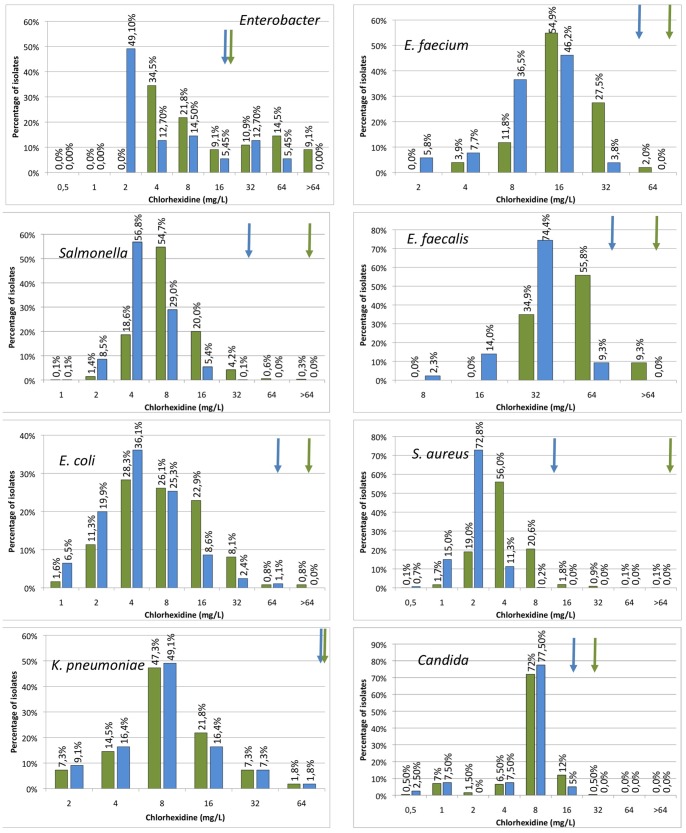
Populational susceptibility to chlorhexidine of different pathogens: To analyze the overall susceptibility to chlorehexidine, the MICs and MBCs (MFCs in the case of Candida) of several independent isolates were established. Blue bars MICs, green bars MBCs/MFCs. ECOFFs are shown with arrows of the corresponding colour.

**Figure 4:**
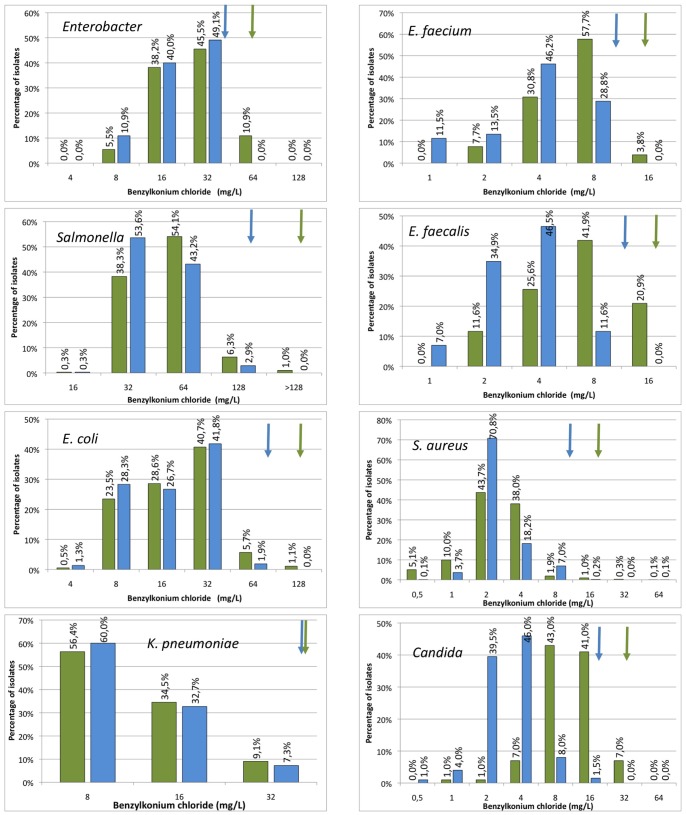
Populational susceptibility to benzalkonium chloride of different pathogens: To analyze the overall susceptibility to benzalkonium chloride, the MICs and MBCs (MFCs in the case of Candida) of several independent isolates were established. Blue bars MICs, green bars MBCs/MFCs. ECOFFs are shown with arrows of the corresponding colour.

**Figure 5:**
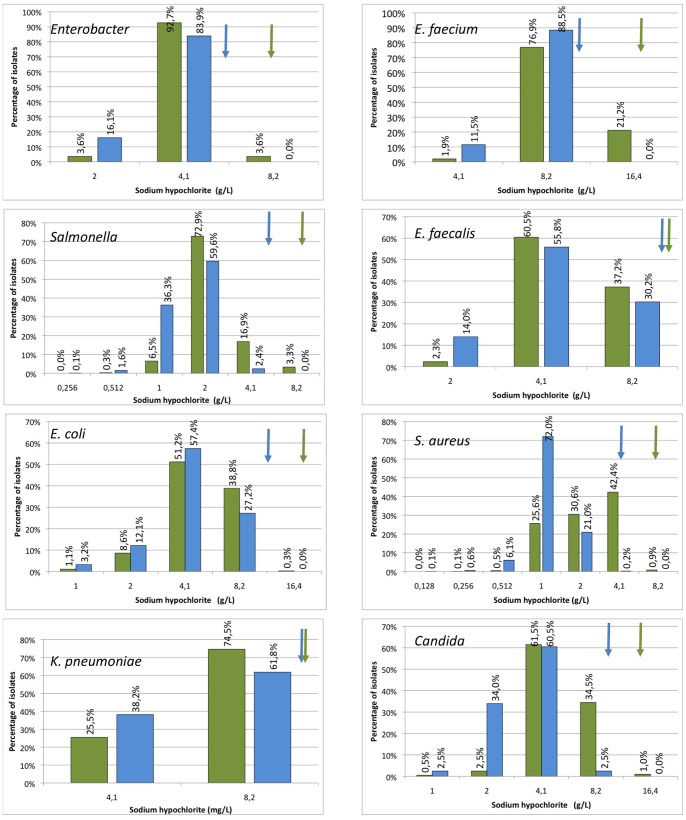
Populational susceptibility to hypochloride of different pathogens: To analyze the overall susceptibility to hypochloride, the MICs and MBCs (MFCs in the case of Candida) of several independent isolates were established. Blue bars MICs, green bars MBCs/MFCs. ECOFFs are shown with arrows of the corresponding colour. Concentration of NaOCl is expressed as active chlorine.

For all other combinations of biocides and micro-organisms, modal distributions, reflecting the lack of clearly resistant subpopulations, were found (Figures 2, 3, 4, 5). This is particular cumbersome in the case of *qac* genes, which are present in widely distributed integrons or plasmids and for which a role on resistance to benzalkonium chloride, and hence on co-selection of resistance determinants present in the mobile elements have been proposed [19], [20]. Bimodal distributions, reflecting the presence of *qac* genes may be predicted. However, these distributions were not found and a correlation between the presence of *qac* determinants and a clear increase in resistance was not fully evident when analyzed ([21], TC *et al.* unpublished). It is worth mentioning that most contemporary studies in this topic are based on correlation analysis on the abundance of integrons containing *qac* genes on contaminated and non-contaminated environments; and not on the phenotype of susceptibility to biocides of the overall microbial populations. Since contaminated ecosystems frequently harbor several different pollutants, including antibiotics, it is difficult to ascertain which is the selective force that selects bacteria carrying integrons in such environments. In this regard, two recent articles indicate that the actual effect of *qac* genes on resistance to quaternary ammonium compounds may be low [22], although they can reduce significantly the susceptibility to other compounds as ethidium bromide that are not used as biocides These results would seem to cast doubts on the actual role that quaternary ammonium-based biocides may have on selection of bacteria carrying integrons containing *qac* and antibiotic resistance genes. Although our results do not preclude the existence of resistant isolates that can be found if more isolates are analyzed, they show that the prevalence of these biocide resistant subpopulations in natural microbial populations is low.

The absence of MIC multimodal distributions indicate that, in sharp contrast with the situation observed for antibiotics, there is no clear evidence that the use of biocides have consistently selected resistant subpopulations presenting MICs above wild-type values, at least by using classical double dilution susceptibility tests. There is an exception to this however; in the case of *S. aureus* susceptibility to TRI a mechanism of resistance acquired by horizontal gene transfer, and rendering a bimodal distribution of triclosan susceptibility, has been recently described [18]. In any case, it is important to mention that TRI resistance in *S. aureus* is due to the heterologous duplication of the gene *fabI*, which encodes the TRI target, and this duplication did not affect the susceptibility to antibiotics currently in use at clinics.

To the best of our knowledge, this is the largest analysis on biocide MICs or MBCs and the only one to determine ECOFFs for biocides. These data provide a baseline to measure biocide susceptibility to assist with future surveillance studies. The finding that in most cases, we did not find bimodal distributions indicates the lack of a relevant percentage of biocide resistant isolates at natural populations. If biocide resistant mutants are rare, this would imply that co-selection or cross-selection of antibiotic resistance should also be a rare event in natural populations.

Nevertheless, two other issues must be taken into consideration. Firstly, most biocides have been widely used for decades; the fact that we did not find bimodal MIC/MBC distributions in current populations may reflect the lack of resistance but also a full replacement of susceptible microorganisms by more resistant ones. This situation that has been named as MIC-creep, which can be defined as “the constant rise over time in the basal intrinsic resistance of an average isolate of a given bacterial species [23]” has been described for different antibiotics [24], [25]. Secondly, our analysis reflects the current steady state of the overall susceptibility to biocides of the studied microbial populations. These observed distributions are the consequence of the emergence of resistance, but also of its spread and stability, the latter being mainly dependent on the fitness costs associated to the acquisition of resistance [26]–[29]. As stated above, several recent studies have shown that microorganisms can evolve to acquire biocide resistance, which in several cases, may be associated to resistance to antibiotics [7], [8], [14]–[16]. Although careful studies on this issue are still scarce [30], it is possible that the stability of these ‘potential’ mechanisms of resistance is impeded by the fitness costs they confer [31]. However, if these mutants are selected at a clinical setting and infect a patient before they are outcompeted by wild-type populations, they can be still a risk for antibiotic therapy.
